# Human Hematopoietic Stem Cells Can Survive In Vitro for Several Months

**DOI:** 10.1155/2009/936761

**Published:** 2008-02-08

**Authors:** Taro Ishigaki, Kazuhiro Sudo, Takashi Hiroyama, Kenichi Miharada, Haruhiko Ninomiya, Shigeru Chiba, Toshiro Nagasawa, Yukio Nakamura

**Affiliations:** ^1^Cell Engineering Division, RIKEN BioResource Center, Tsukuba, Ibaraki 305-0074, Japan; ^2^Division of Hematology, Institute of Clinical Medicine, University of Tsukuba, Tsukuba, Ibaraki 305-8575, Japan

## Abstract

We previously reported that long-lasting in vitro hematopoiesis could be achieved using the cells differentiated from primate embryonic stem (ES) cells. Thus, we speculated that hematopoietic stem cells differentiated from ES cells could sustain long-lasting in vitro hematopoiesis. To test this hypothesis, we investigated whether human hematopoietic stem cells could similarly sustain long-lasting in vitro hematopoiesis in the same culture system. Although the results varied between experiments, presumably due to differences in the quality of each hematopoietic stem cell sample, long-lasting in vitro hematopoiesis was observed to last up to nine months. Furthermore, an in vivo analysis in which cultured cells were transplanted into immunodeficient mice indicated that even after several months of culture, hematopoietic stem cells were still present in the cultured cells. To the best of our knowledge, this is the first report to show that human hematopoietic stem cells can survive in vitro for several months.

## 1. Introduction

Identification of in vitro culture protocols that enable somatic stem cells to survive and proliferate will be of value not only for basic research but also clinical applications that require somatic stem cells. The development of an efficient method for in vitro proliferation of mesenchymal stem cells, for example, has allowed cultured mesenchymal stem cells to be used in clinical applications [1].

Although hematopoietic stem cells have been extensively analyzed and characterized [2], in vitro proliferation of these cells remains problematic using established culture methods [3, 4]. In addition, the length of time that hematopoietic stem cells can survive in an in vitro culture system remains uncertain. CD34-positive (CD34^+^) cells have been identified in long-term in vitro cultures of hematopoietic stem cells [5–9]. However, as none of these previous studies performed an in vivo assay of the cultured cells, such as transplantation into mice, it is uncertain whether hematopoietic stem cells with the capacity to reconstitute long-term in vivo hematopoiesis were present in these prolonged in vitro cultures.

We previously described a culture method that produced long-lasting in vitro hematopoiesis using non-human primate embryonic stem (ES) cells [10]. We speculated that hematopoietic stem cells derived from ES cells could sustain long-lasting in vitro hematopoiesis. To test this hypothesis, we initiated long-term in vitro cultures of human hematopoietic stem cells using the same culture method as previously [10]. In addition, we evaluated the in vivo function of cells cultured in vitro for several months by transplanting them into immunodeficient mice.

## 2. Materials and Methods

### 2.1. Cell Culture

We purchased human umbilical cord blood samples from the Cell Engineering Division of RIKEN BioResource Center (Tsukuba, Ibaraki, Japan). The ethical committee of the RIKEN Tsukuba Institute approved the use of human umbilical cord blood before the study was initiated. CD34^+^ hematopoietic stem/progenitor cells were collected from human umbilical cord blood using a magnetic cell sorting system, MACS CD34 Isolation kit (Miltenyi Biotec Inc., Sunnyvale, Calif, USA), according to the manufacturer's instructions.

Mouse-derived cell lines (OP9 and C3H10T1/2) were purchased from the Cell Engineering Division of RIKEN BioResource Center (Tsukuba, Ibaraki, Japan) and were cultured as follows: OP9 in Minimum Essential Medium-*α* (MEM-*α*; Invitrogen, Carlsbad, Calif, USA) containing 20% fetal bovine serum (FBS; Invitrogen, Calif, USA); C3H10T1/2 in Basal Medium Eagle (BME; Invitrogen) containing 10% FBS (BioWest, Miami, Fla, USA). The cell lines were *γ*-irradiated (50 Gy) before use as feeder cells.

CD34^+^ cells were cultured on feeder cells in a 100 mm dish in Iscove's modified Dulbecco's medium (IMDM; SIGMA, St Louis, Mo, USA) containing 10% FBS (BioWest), 10 *μ*g/mL bovine insulin, 5.5 *μ*g/mL human transferrin, 5 ng/mL sodium selenite (ITS liquid MEDIA supplement; SIGMA-Aldrich, Mass, USA), 100 unit/mL penicillin, 100 *μ*g/mL streptomycin, 2 mm L-glutamine (PSQ; Invitrogen), 50 ng/mL stem cell factor (SCF; R&D Systems, Minneapolis, Minn, USA), 50 ng/mL Flt-3 ligand (Flt-3L; R&D Systems), and 50 ng/mL thrombopoietin (TPO; R&D Systems). The initial number of CD34^+^ cells placed in culture varied between experiments: 5 × 10^3^ cells in Exp-OP9-A, Exp-10T1/2-A, and Exp-OP9-B; 4 × 10^4^ cells in Exp-OP9-F and Exp-10T1/2-F; 5 × 10^4^ cells in Exp-OP9-C, Exp-OP9-E, Exp-10T1/2-E, Exp-OP9-H, and Exp-10T1/2-H; 8 × 10^4^ cells in Exp-OP9-G and Exp-10T1/2-G; 1 × 10^5^ cells in Exp-10T1/2-I; 2 × 10^5^ cells in Exp-OP9-D and Exp-10T1/2-D. The letters “A” to “I” after Exp-OP9 or Exp-10T1/2 indicate 9 different umbilical cord blood samples derived from 9 different neonates. Samples A, B, C, F, G, H, and I were frozen after collection, while samples D and E were used immediately as fresh samples.

Twenty-four hours after initiation of culture, the medium together with any detached cells was removed and fresh medium was added to the culture. Thereafter, the medium was changed every 3-4 days (twice a week). The number of cells attached to the feeder cells increased gradually. Approximately four weeks after initiation of culture, attached cells were dissociated using a 0.25% trypsin EDTA solution (SIGMA-Aldrich) and cultured again on new feeder cells. Thereafter, similar passages of attached cells were performed every 3-4 weeks.

The number of viable cells was assessed using an automated cell counter and an assay based on the trypan blue dye exclusion method, ViCell (BECKMAN COULTER, Fullerton, Calif, USA). The morphology of the cells was analyzed by microscopic examination after Wright staining (Muto Pure Chemicals, Tokyo, Japan).

### 2.2. Flow Cytometry

Cells were stained with monoclonal antibodies (MoAbs) and analyzed using a FACS Calibur (BD Biosciences, San Jose, Calif, USA). The following MoAbs were purchased from BD Biosciences: fluorescein isothiocyanate- (FITC-) conjugated MoAb against human CD14 (FITC-CD14), FITC-CD34, FITC-CD41a, and FITC-CD45; phycoerythrin-conjugated MoAb against human CD4(PE-CD4), PE-CD11b, PE-CD13, PE-CD34, PE-CD45, PE-CD56, and PE-CD235a (Glycophorin A); allophycocyanin-conjugated MoAb against human CD3 (APC-CD3), APC-CD8, APC-CD19, APC-CD33, and APC-CD45. PE-CD33 was purchased from eBiosciences (San Diego, Calif, USA). FITC-mouse IgG1, PE-mouse IgG1, APC-mouse IgG1, FITC-mouse IgG2a, and PE-mouse IgG2b were also purchased from BD Biosciences and were used as isotype controls. Cell viability was monitored by staining with propidium iodide (SIGMA-Aldrich). Flow cytometry data were analyzed using CellQuest (BD Biosciences) analysis software.

### 2.3. Colony Formation Assay

Cells (1 × 10^4^) were cultured in a 35 mm dish with 1 mL of Methocult (H4435; Stem cell technology, Vancouver, British Columbia, Canada) for 10–14 days, and the number of separate colonies was determined by macroscopic morphology. Representative colonies were picked up and cell morphology was analyzed by microscopic examination after Wright staining (Muto Pure Chemicals).

### 2.4. Cell Transplantation into Mice

Eight-week-old male NOD/Shi-*scid* IL-2R*γ* ^null^ (NOG) mice were purchased from the Central Institute for Experimental Animals (Kawasaki, Kanagawa, Japan) and used within two weeks of delivery in all experiments. Prior to cell transplantation, the mice were given a sublethal dose of *γ*-rays (3.0 Gy). A 200 *μ*L cell suspension in phosphate-buffered saline (PBS; SIGMA) containing 5% FBS (BioWest) was injected intravenously into the tail vein of each mouse. All experimental procedures on the mice were approved by the Institutional Animal Care and Use Committee of the RIKEN Tsukuba Institute.

## 3. Results and Discussion

### 3.1. Long-Lasting In Vitro Hematopoiesis from Human Hematopoietic Stem Cells

Human CD34^+^ cells were cultured on feeder cells in the presence of SCF, Flt-3L, and TPO. We used the mouse-derived cell lines, OP9 and C3H10T1/2, as feeder cells. Both OP9 [10–15] and C3H10T1/2 [10, 16–18] have been used in many studies to maintain in vitro hematopoiesis. As we show below, both OP9 and C3H10T1/2 cells supported long-lasting in vitro hematopoiesis from human hematopoietic stem cells.

**Figure 1:**
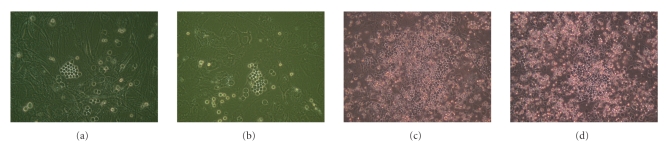
Appearance of the cells attached to feeder cells. Representative examples of CD34(+) human hematopoietic stem/progenitor cells cultured on either OP9 feeder cells ((a), (c)) or C3H10T1/2 feeder cells ((b), (d)) for 7 days ((a), (b)) or 22 days ((c), (d)).

About one week after initiation of culture, cobblestone areas were observed on the feeder cells (Figures 1(a) and 1(b)), indicating that human hematopoietic cells were proliferating. The numbers of cells attached to the feeder cells increased gradually (Figures 1(c) and 1(d)) and the numbers of detached cells, derived from the attached cells, also increased gradually. When the medium was changed, the numbers of detached cells in the medium were counted and the cells were subjected to analyses such as flow cytometric analysis. Detached cells were removed during the medium changes and were not cultured further in any of the experiments.

**Figure 2:**
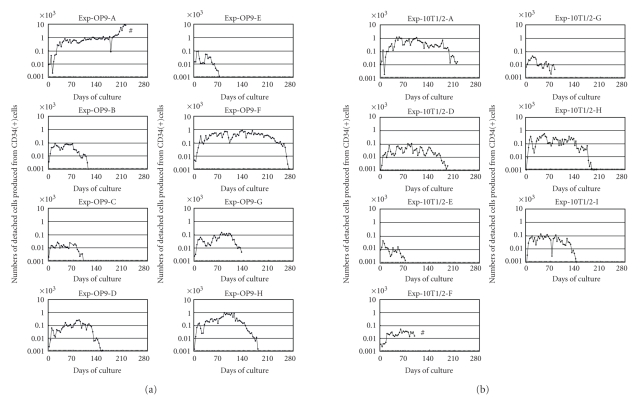
Production of hematopoietic cells in long-term cultures of human hematopoietic stem cells. (a) Eight independent experiments were performed using eight different umbilical cord blood samples and OP9 cells as feeder cells. (b) Seven independent experiments were performed using seven different umbilical cord blood samples and C3H10T1/2 cells as feeder cells. ((a), (b)) The number of detached cells in the overlying medium was counted at each medium change (approximately half weekly). The data are shown as the mean number of detached cells produced from a single CD34(+) cell, that is, the total number of detached cells divided by the number of CD34(+) cells used to initiate the culture. Exp: experiment. A to I after Exp-OP9 and Exp-10T1/2 indicate 9 different umbilical cord blood samples derived from 9 different neonates. #: Cultures Exp-OP9-A and Exp-10T1/2-F were terminated because of fungal infection.

Detached cells were continuously produced for several months in experiments Exp-OP9-A, Exp-OP9-F, Exp-OP9-H, Exp-10T1/2-A, and Exp-10T1/2-H (Figure 2). As we detail below, flow cytometric analysis and a transplantation assay demonstrated that the detached cells produced in this culture method included both mature and immature hematopoietic cells, such as colony-forming cells and hematopoietic stem cells. We found that production of detached cells eventually ceased in all experiments except for Exp-OP9-A. Unfortunately, we were forced to halt Exp-OP9-A because of fungal infection although the cells in this culture proliferated efficiently and robustly before fungal contamination (Figure 2).

The numbers of detached cells varied among the experiments (Figure 2) and, notably, showed no correlation with the initial number of CD34^+^ cells used in each experiment. Thus, 5 × 10^3^ CD34^+^ cells were used in Exp-OP9-A and Exp-10T1/2-A and both cultures produced substantial numbers of detached cells over a prolonged period (Figure 2). In contrast, a larger number of CD34^+^ cells (2 × 10^5^) was used to initiate the Exp-OP9-D and Exp-10T1/2-D cultures, but they produced considerably fewer detached cells (Figure 2). These results indicate that the rate of production of detached cells depended on the quality rather than the number of CD34^+^ cells used in each experiment. In other words, when the quality of CD34^+^ cells was high, 5 × 10^3^ CD34^+^ cells were sufficient to generate efficient in vitro hematopoiesis as shown in Exp-OP9-A and Exp-10T1/2-A (Figure 2).

**Figure 3:**
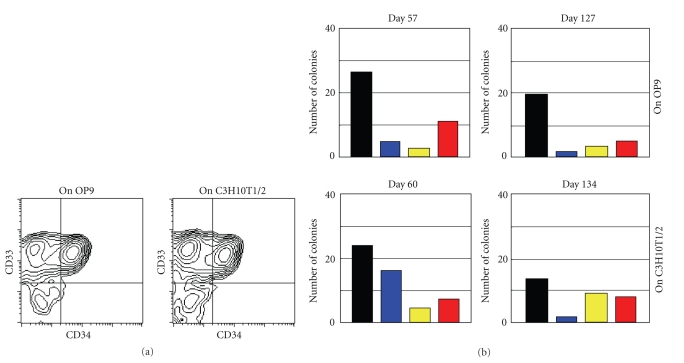
Characterization of cultured cells. (a) Flow cytometric analysis of detached cells produced in cultures on OP9 (Exp-OP9-A) and C3H10T1/2 (Exp-10T1/2-A) feeder cells and collected on Day 218 of culture. The detached cells were stained for CD33, a marker specific for granulocyte/macrophage lineage cells, and CD34, a marker specific for hematopoietic stem/progenitor cells. Flow cytometric analyses of detached cells from other experiments showed similar results. (b) Colony-formation assays. Detached cells produced in culture on OP9 feeder cells (Exp-OP9-A) were collected on Days 57 and 127 of culture. Similarly, detached cells produced in culture on C3H10T1/2 feeder cells (Exp-10T1/2-D) were collected on Days 60 and 134 of culture. The cell samples were used in a standard colony-formation assay. Black bars: colony-forming unit of monocyte/macrophage lineage cells, CFU-M. Blue bars: colony-forming unit of granulocyte lineage cells, CFU-G. Yellow bars: colony-forming unit of granulocyte and monocyte/macrophage lineage cells, CFU-GM. Red bars: burst-forming unit of erythroid cells, BFU-E. Similar results were obtained in colony-formation assays using detached cells from other cultures.

The majority of detached cells had the morphological characteristics of granulocyte/macrophage lineage cells (Supplementary Figure S1, available at doi 10.1155/2009/936761) although some blast-like cells were also present. Consistent with their morphological phenotype, the majority of the detached cells expressed CD33, a marker of granulocyte/macrophage lineage cells (Figure 3(a)). Of note, CD34^+^CD33^+^ cells, which are less mature than CD34^−^CD33^+^ cells, were abundant among the detached cells even at 7 months after initiation of culture (Figure 3(a)).

A colony-formation assay demonstrated that granulocyte, macrophage, and erythrocyte progenitor cells were present among the detached cells (Figure 3(b)). As mentioned above, when the culture medium was changed, detached cells were removed and were not cultured further. Instead, they were either used in experimental analyses or discarded. As is shown in Figure 2, detached cells were continuously produced, and they included abundant colony-forming cells even at Day 127 and Day 134. As one example, the calculated total number of colony-forming cells present in detached cells at Day 127 (upper right, Figure 3(b)) was 13 311, which corresponded to a greater than ten-fold increase in the numbers of colony-forming cells compared to the starting material of this culture, that is, 983 colony-forming cells in 5 × 10^3^ CD34^+^ cells. Thus, the culture method we describe here could continuously produce abundant colony-forming cells for several months.

Although a mixed colony (a colony derived from very immature hematopoietic cells) was not observed in the colony formation assay (Figure 3(b)), it nevertheless remained possible that hematopoietic stem cells were present at a very low frequency among the detached cells. Therefore, we performed a transplantation assay in which detached cells were injected into an immunodeficient NOD/Shi-*scid* IL-2R*γ* ^null^ (NOG) mouse (mentioned hereafter).

### 3.2. Hematopoietic Stem Cells Cultured In Vitro for Several Months Give Rise to Long-Lasting In Vivo Hematopoiesis after Transplantation into Mice

**Figure 4:**
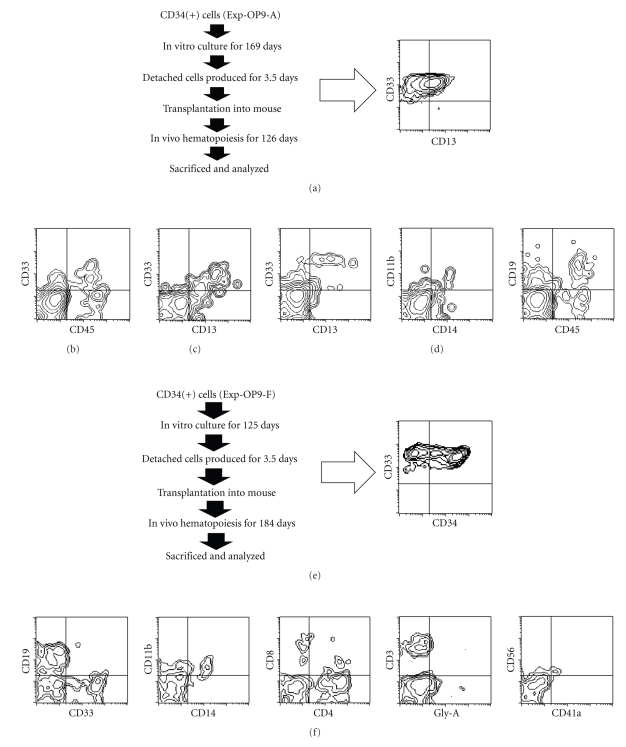
Flow cytometric analysis of hematopoietic cells of the mouse that had been transplanted with human hematopoietic cells produced by in vitro culture. ((a), (e)) Schema of the experimental procedure and flow cytometric analysis of transplanted cells. ((a)–(d)) Detached cells (3.9 × 10^6^ cells) produced on OP9 feeder cells (Exp-OP9-A) were collected on Day 169 of culture and transplanted into an immunodeficient NOG mouse. Peripheral blood was collected on Days 56 (b) and 112 (c) after transplantation, and bone marrow cells were collected on Day 126 after transplantation (d). ((e), (f)) Detached cells (2.4 × 10^6^ cells) produced on OP9 feeder cells (Exp-OP9-F) were collected on day 125 of culture and transplanted into an immunodeficient NOG mouse. The bone marrow cells were collected on Day 184 after transplantation and were analyzed. ((a)–(f)) The cells were stained using monoclonal antibodies against CD45, a leukocyte common antigen, CD34, a marker specific for hematopoietic stem/progenitor cells, CD33 and CD13, markers of granulocyte and monocyte/macrophage lineage cells, CD11b and CD14, markers of monocyte/macrophage lineage cells, CD19, a marker of B lymphocyte lineage cells, CD3, CD4, and CD8, markers of T lymphocyte lineage cells, Gly-A (Glycophorin A), a marker of erythroid cells, CD56, a marker of large granular lymphocytes and natural killer cells, and CD41a, a marker of megakaryocyte/platelet lineage cells.

Detached cells were collected from Exp-OP9-A on Day 169 after initiation of culture and transplanted into an NOG mouse (3.9 × 10^6^ cells) (Figure 4(a)). Peripheral blood samples from the mouse were subjected to flow cytometric analysis on Days 56 and 112 after transplantation. Human hematopoietic cells were clearly present in the peripheral bloods (Figures 4(b), 4(c)). The mouse was sacrificed on Day 126 after transplantation, and bone marrow and spleen cells were subjected to flow cytometric analysis. Human hematopoietic cells were present in the bone marrow (Figure 4(d)) but were present at a very low rate in spleen (data not shown). The estimated rate of chimerism of human CD45^+^ cells in bone marrow was 2.6% when compared to the number of mouse CD45^+^ cells. The human hematopoietic cells detected in the bone marrow included cells of the myeloid lineage (CD13^+^CD33^+^: 9.7% of the human CD45^+^ cells), the monocyte/macrophage lineage (CD11b^+^CD14^+^: 3.8% of the human CD45^+^ cells), the B cell lineage (CD19^+^: 80.5% of the human CD45^+^ cells) (Figure 4(d)), and other lineages at very low levels (data not shown).

Detached cells were collected from Exp-OP9-F on day 125 after initiation of culture and transplanted into an NOG mouse (2.4 × 10^6^ cells) (Figure 4(e)). CD45^+^ human hematopoietic cells were present in peripheral blood from the mouse one month after transplantation (data not shown). The mouse was sacrificed on Day 184 after transplantation, and the bone marrow cells were subjected to flow cytometric analysis. The rate of chimerism of human CD45^+^ cells in the bone marrow was 0.2% when compared to the number of mouse CD45^+^ cells. The bone marrow contained human hematopoietic cells, which included cells of the myeloid lineage (CD33^+^: 23.5% of the human CD45^+^ cells), the monocyte/macrophage lineage (CD11b^+^ CD14^+^: 6.7% of the human CD45^+^ cells), as well as the B (CD19^+^: 53.7% of the human CD45^+^ cells) and T cell lineages (CD3^+^, CD4^+^, CD8^+^, and CD4^+^CD8^+^: 10.7% of the human CD45^+^ cells) (Figure 4(f)). Erythroid (Glycophorin A^+^) and megakaryocyte (CD41a^+^) lineage cells were present at very low rates (Figure 4(f)).

In the transplantation assay described above, human hematopoietic cells of various lineages were present in mice up to six months after cell transplantation. In general, it is impossible that in vivo hematopoiesis derived from transplanted cells is maintained for several months solely by committed progenitor cells. Therefore, the transplanted cells appeared to include hematopoietic stem cells, that is, even after in vitro culture for several months, hematopoietic stem cells were still present in cultures Exp-OP9-A and Exp-OP9-F. In light of the numbers of cells produced in culture and of the duration of this production, the hematopoietic stem cells also appeared to have survived for several months in Exp-OP9-H, Exp-10T1/2-A, and Exp-10T1/2-H.

The in vitro expansion of human hematopoietic stem cells is known to be very difficult [3]. In agreement with this, a mixed colony (a colony derived from very immature hematopoietic cells) was not observed in the colony formation assay in this study (Figure 3(b)), indicating that hematopoietic stem cells did not expand to any great extent in our culture method. However, since it was highly unlikely that in vitro hematopoiesis could be maintained for several months solely by committed progenitor cells present in the starting materials, the long-lasting in vitro hematopoiesis was likely maintained by hematopoietic stem cells.

On the basis of a previous estimation of the numbers of hematopoietic stem cells capable of repopulating in NOD/SCID mice [19], the starting materials we used in Exp-OP9-A and Exp-OP9-F should have included a very low number of NOD/SCID-repopulating cells. However, the transplantation assay demonstrated that NOD/SCID-repopulating cells were present among the detached cells that were continuously produced in our culture method (Figure 4), strongly suggesting that our culture method continuously produced small numbers of new NOD/SCID-repopulating cells throughout the long-term culture period. Hence, the total number of NOD/SCID-repopulating cells that were produced as detached cells throughout the whole long-term in vitro culture was likely greater than the number of such cells that were present in the starting materials.

Taken together, the hematopoietic stem cells capable of repopulating in NOD/SCID mice in our culture system appeared to be maintained by asymmetric cell division, that is, one of the daughter cells retained the characteristics of hematopoietic stem cells and another did not.

Leukemic stem cells might also survive and/or proliferate in our culture method for a prolonged period, enabling basic research or screening for effective anticancer drugs to be performed on these cultured cells. In addition, basic research on specific diseases, such as aplastic anemia or paroxysmal nocturnal hemoglobinuria, might benefit from a long-term culture system for hematopoietic stem cells derived from patients.

## 4. Conclusions

To the best of our knowledge, this is the first report to show that human hematopoietic stem cells can survive in vitro for several months. Since the duration of in vitro hematopoiesis appeared to depend on the quality of hematopoietic stem cells present in each sample, our culture method may be of value for assessing the quality of hematopoietic stem cells prior to their use in the clinic. In particular, our method could be used for the evaluation of umbilical cord bloods since these samples are routinely used in the clinic following preservation for several months. For example, in this study the quality of hematopoietic stem cells derived from samples A, F, and H appeared to be higher compared to other samples.

## Supplementary Material

Supplementary Figure S1 shows that the majority of detached cells had the morphological characteristics of granulocyte/macrophage lineage cells.
