# Supplementary material for: Human Hematopoietic Stem Cells Can Survive In Vitro for Several Months
Source: Adv Hematol. 2008 Feb 8;2009:936761. doi: 10.1155/2009/936761 (PMC2778179; doi:10.1155/2009/936761)
Supplement: Supplementary file 1 — Supplementary Figure S1 shows that the majority of detached cells had the morphological characteristics of granulocyte/macrophage lineage cells. [file 936761.f1.pdf]

Supplementary Figure S1

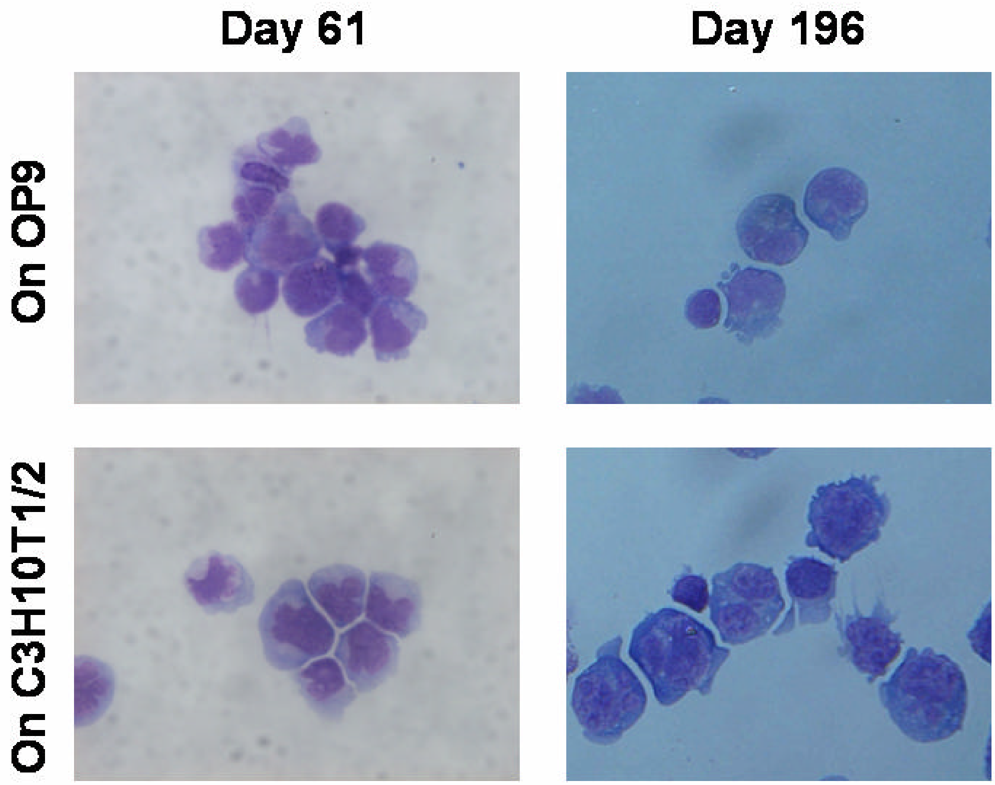

Supplementary Figure S1. Morphology of detached cells. The images show detached cells from cultures on OP9 (Exp-OP9-A) or C3H10T1/2 (Exp-10T1/2-A) feeder cells collected on days 61 and 196 of culture. The cells are Wright stained. The morphology of detached cells from other experiments was similar to those shown here.
